# Transgene silencing of sucrose synthase in alfalfa (*Medicago sativa* L.) stem vascular tissue suggests a role for invertase in cell wall cellulose synthesis

**DOI:** 10.1186/s12870-015-0649-4

**Published:** 2015-12-01

**Authors:** Deborah A. Samac, Bruna Bucciarelli, Susan S. Miller, S. Samuel Yang, Jamie A. O’Rourke, Sanghyun Shin, Carroll P. Vance

**Affiliations:** USDA-ARS-Plant Science Research Unit, St. Paul, MN 55108 USA; Department of Plant Pathology, University of Minnesota, St. Paul, MN 55108 USA; Department of Agronomy and Plant Genetics, University of Minnesota, St. Paul, MN 55108 USA; Present address: Monsanto Company, Chesterfield, MO 63017 USA; Present address: USDA-ARS-Corn Insects and Crop Genetics Research Unit, Ames, IA 50011 USA; Present address: National Institute of Crop Science, Iksan, 570-080 Korea

**Keywords:** Biofuels, Cell wall biosynthesis, Cellulose, Gene silencing, Phloem, Xylem

## Abstract

**Background:**

Alfalfa (*Medicago sativa* L.) is a widely adapted perennial forage crop that has high biomass production potential. Enhanced cellulose content in alfalfa stems would increase the value of the crop as a bioenergy feedstock. We examined if increased expression of sucrose synthase (SUS; EC 2.4.1.13) would increase cellulose in stem cell walls.

**Results:**

Alfalfa plants were transformed with a truncated alfalfa phosphoenolpyruvate carboxylase gene promoter (PEPC7-P4) fused to an alfalfa nodule-enhanced SUS cDNA (*MsSUS1*) or the β-glucuronidase (*GUS*) gene. Strong GUS expression was detected in xylem and phloem indicating that the PEPC7-P4 promoter was active in stem vascular tissue. In contrast to expectations, *MsSUS1* transcript accumulation was reduced 75–90 % in alfalfa plants containing the *PEPC7-P4::MsSUS1* transgene compared to controls. Enzyme assays indicated that SUS activity in stems of selected down-regulated transformants was reduced by greater than 95 % compared to the controls. Although SUS activity was detected in xylem and phloem of control plants by *in situ* enzyme assays, plants with the *PEPC7-P4::MsSUS1* transgene lacked detectable SUS activity in post-elongation stem (PES) internodes and had very low SUS activity in elongating stem (ES) internodes. Loss of SUS protein in PES internodes of down-regulated lines was confirmed by immunoblots. Down-regulation of SUS expression and activity in stem tissue resulted in no obvious phenotype or significant change in cell wall sugar composition. However, alkaline/neutral (A/N) invertase activity increased in SUS down-regulated lines and high levels of acid invertase activity were observed. *In situ* enzyme assays of stem tissue showed localization of neutral invertase in vascular tissues of ES and PES internodes.

**Conclusions:**

These results suggest that invertases play a primary role in providing glucose for cellulose biosynthesis or compensate for the loss of SUS1 activity in stem vascular tissue.

**Electronic supplementary material:**

The online version of this article (doi:10.1186/s12870-015-0649-4) contains supplementary material, which is available to authorized users.

## Background

Alfalfa (*Medicago sativa* L.) is the most widely grown forage legume across the globe and plays key roles in livestock nutrition, protecting water and soil resources, enhancing soil fertility, and sequestering soil carbon. In addition, alfalfa has many attributes that make it attractive as a biofuel feedstock including high biomass yield potential. Due to biological nitrogen fixation, alfalfa usually requires no nitrogen fertilizer, and can provide all of the nitrogen required for the following grain crop. Alfalfa forage can be fractionated into protein-rich leaves and cellulose-rich stems to create two product streams. The stems can be used for production of energy by fermentation to ethanol or gasification to produce electricity. Developing varieties with increased cellulose would enhance the value of alfalfa as a cellulosic biomass feedstock [1]. All tissues in elongating stem internodes (except protoxylem vessel cells) deposit thin, cellulose-poor primary cell walls [2, 3]. In contrast, thick, cellulose-rich secondary walls are deposited in phloem and xylem fiber cells in post-elongation stem internodes. One strategy for increasing cellulose is to increase the expression of enzymes involved in cellulose synthesis in vascular cells in alfalfa stems.

Sucrose synthase (SUS; EC 2.4.1.13), a glycosyltransferase that catalyzes the reversible conversion of sucrose into fructose and UDP-glucose, has been thought to play a major role in providing UDP-glucose for cellulose synthesis [4–6]. SUS is encoded by a small gene family in most plant species [7–11]. In *M. truncatula*, a close relative of alfalfa, five *SUS* genes were identified [11] and six isoforms were identified in the model legume *Lotus japonicus* [12]. In alfalfa, less is known about the *SUS* gene family. Currently, only one *SUS* gene sequence, for the *MsSUS1* isoform, is present in GenBank (AF049487). In several plants, an increase in SUS expression was correlated with an increase in cellulose [13, 14]. The over-expression of an *SUS* gene from cotton (*Gossypium hirsutum* L.) in hybrid poplar under the control of either the *cauliflower mosaic virus* 35S promoter or a xylem-specific promoter increased SUS enzyme activity and cellulose in secondary xylem [15].

Sucrose is also hydrolyzed into glucose and fructose by invertase enzymes. Invertases are classified into two major groups, the acid invertases, located primarily in the cell wall and vacuole, and the alkaline/neutral (A/N) invertases located in the cytosol, mitochondria, and plastids [16]. Invertases were thought to have a minor role in sucrose metabolism, but recent studies have shown them to potentially have a broader role in sucrose catabolism. Mutated *Arabidopsis thaliana* plants lacking four of the six isoforms of *SUS* (*sus1/sus2/sus3/sus4*) and reported to lack soluble and membrane bound SUS activity, nonetheless exhibited normal growth and cellulose content. However, mutation of two neutral cytosolic invertase isoforms (*cinv1/cinv2*) resulted in severe inhibition of growth [17]. Similarly, mutation of the predominant isoform of cytosolic invertase in *L. japonicus, LjINV1*, resulted in a severe reduction in growth of roots and shoots, a change in cellular development, and impaired flowering [18]. However, mutation of the predominant SUS isoforms, *LjSUS1* and *LjSUS3*, had little effect on plant growth, reproduction, or nitrogen fixation. Only when the *sus1-1/sus3-1* double mutant was grown in the absence of nitrogen was leaf number and shoot weight reduced compared to wild-type plants [12].

Previous work showed that an alfalfa phosphoenolpyruvate carboxylase gene (*PEPC-7*) was expressed at high levels in alfalfa root nodules [19]. The full-length promoter (−1299 to +86 relative to the transcription initiation site) fused to the β-glucuronidase (*GUS*) gene resulted in GUS expression in the root nodule, root tip, and pulvinus. A shorter promoter segment designated P4 (−536 to +86) directed very strong GUS activity in vascular tissue throughout the plant. In stems, GUS activity was localized primarily to xylem cells [20].

We utilized the P4 promoter of *PEPC-7* (PEPC7-P4) to express the *MsSUS1* cDNA in transgenic alfalfa to test the hypothesis that vascular-enhanced expression would increase stem cell wall cellulose. The results showed that in contrast to expectations, expressing *MsSUS1* using the PEPC7-P4 promoter resulted in strong down-regulation of *MsSUS1* transcripts, and eliminated most of the SUS enzyme activity in vascular tissue of alfalfa stems. Down-regulation of SUS had only minor effects on plant growth and cell wall sugar composition of stems. Although SUS activity was very low in transformants with the *PEPC7-P4:: MsSUS1* construct, acid invertase activity was maintained and A/N invertase activity increased. We discuss the implications of the relationship between SUS and invertase, the *SUS* isoforms, and their potential roles in cell wall biosynthesis.

## Results

### Histochemical analysis of the *PEPC7-P4::GUS* reporter

**Fig. 1:**
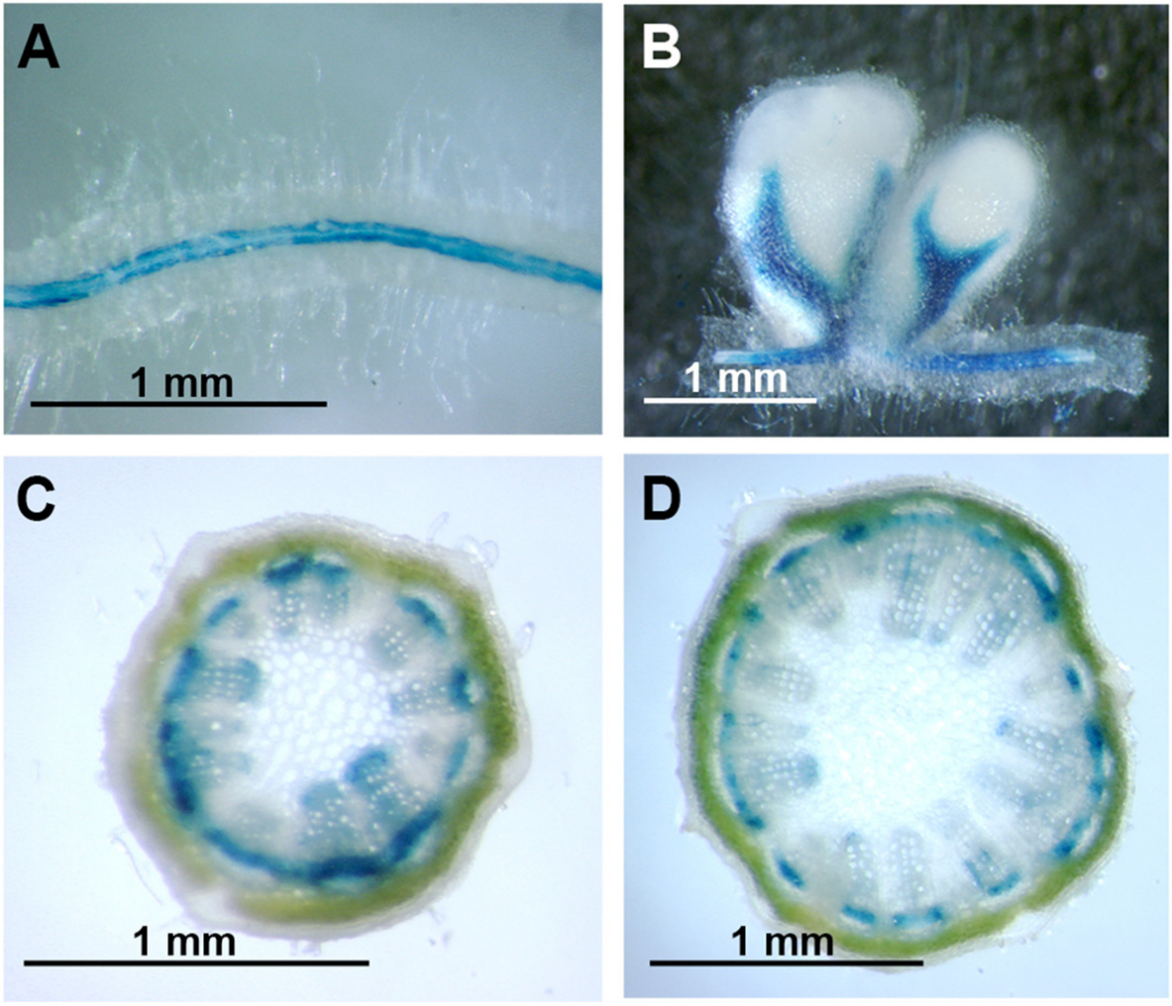
Histochemical GUS staining of alfalfa stem tissue expressing the *PEPC7-P4::GUS* construct. **a**, root; (**b**), nodule; (**c**), transverse section of elongating stem (ES) internode; (**d**), transverse section of post-elongation stem (PES) internode. Scale bar represents 1 mm

Previous work demonstrated that the PEPC7-P4 promoter was active in the vascular tissue of alfalfa nodules, roots, and stems [19, 20]. We examined the GUS staining pattern in nodules, roots, and stems of *PEPC7-P4::GUS* transformants with a more detailed analysis of stem tissues. Our results confirm expression of GUS in vascular tissues of roots and nodules containing the *PEPC7-P4::GUS* construct (Fig. 1a, b). In stems, we found that the PEPC7-P4 promoter was active in both xylem and phloem tissue. GUS staining in phloem was evident in both elongating stem (ES) and post-elongation stem (PES) internodes (Fig. 1c, d). GUS staining in xylem was more evident in ES relative to PES internodes and was primarily localized to the protoxylem and the xylem parenchyma. In apical regions of ES internodes (first and second internodes), GUS staining occurred only in xylem suggesting that the PEPC7-P4 promoter was not active in the protophloem. The transformants selected to be used as transgenic control lines (M22, M35) contained the *PEPC7-P4::GUS* construct but lacked GUS expression as tested by histochemical staining.

### Relative expression of *MsSUS1* in transgenic plants

**Fig. 2:**
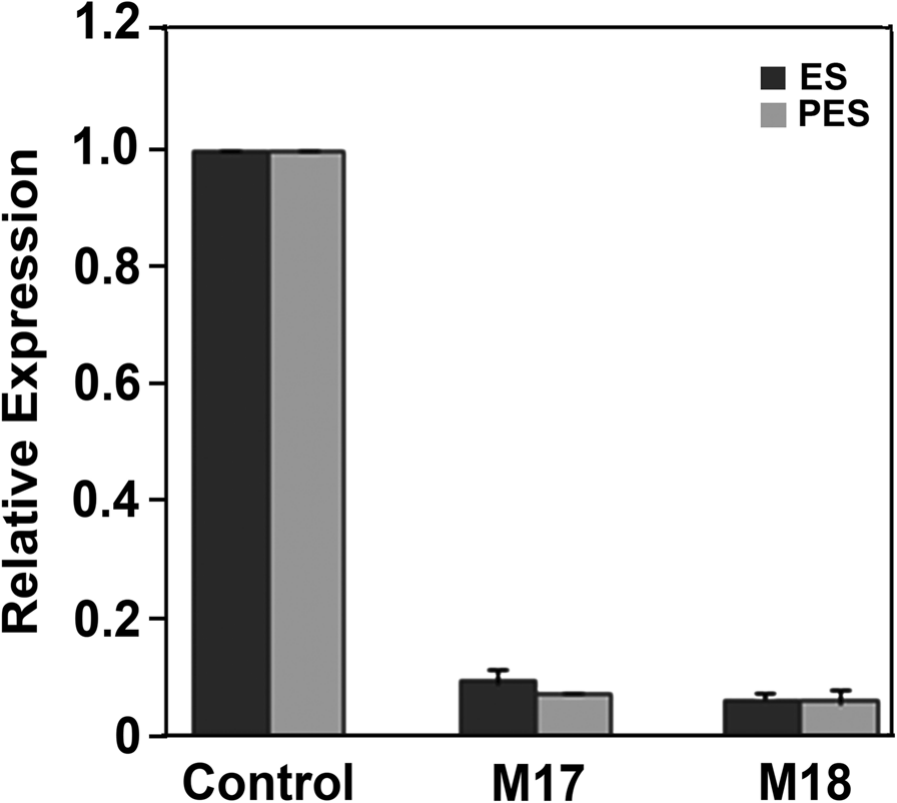
Quantitative reverse transcriptase-PCR of *MsSUS1* transcripts in elongating stem (ES) and post-elongation stem (PES) internodes.Expression values in *MsSUS1* transformants (M17, M18) were calculated relative to *SUS* transcripts in controls. Control values represent the average of two plant lines (M22, M35). Values for M17 and M18 represent means ± standard error (*n* = 3)

Primers specific to the *MsSUS1* transcript were used in quantitative reverse transcriptase PCR (qRT-PCR) assays to measure *MsSUS1* transcript accumulation in stems. A survey of ES internodes from 20 independent *PEPC7-P4::SUS1* transformed lines showed that the *MsSUS1* transcript was reduced 75 to 90 % compared to the mean transcript level in ES internodes of the control lines (M22, M35). Two transformed lines (M17, M18) that exhibited approximately 90 % down-regulation of the *MsSUS1* transcript in both ES and PES internodes compared to the controls (Fig. 2) were selected for further study.

### SUS enzyme and *in situ* enzyme activity

**Fig. 3:**
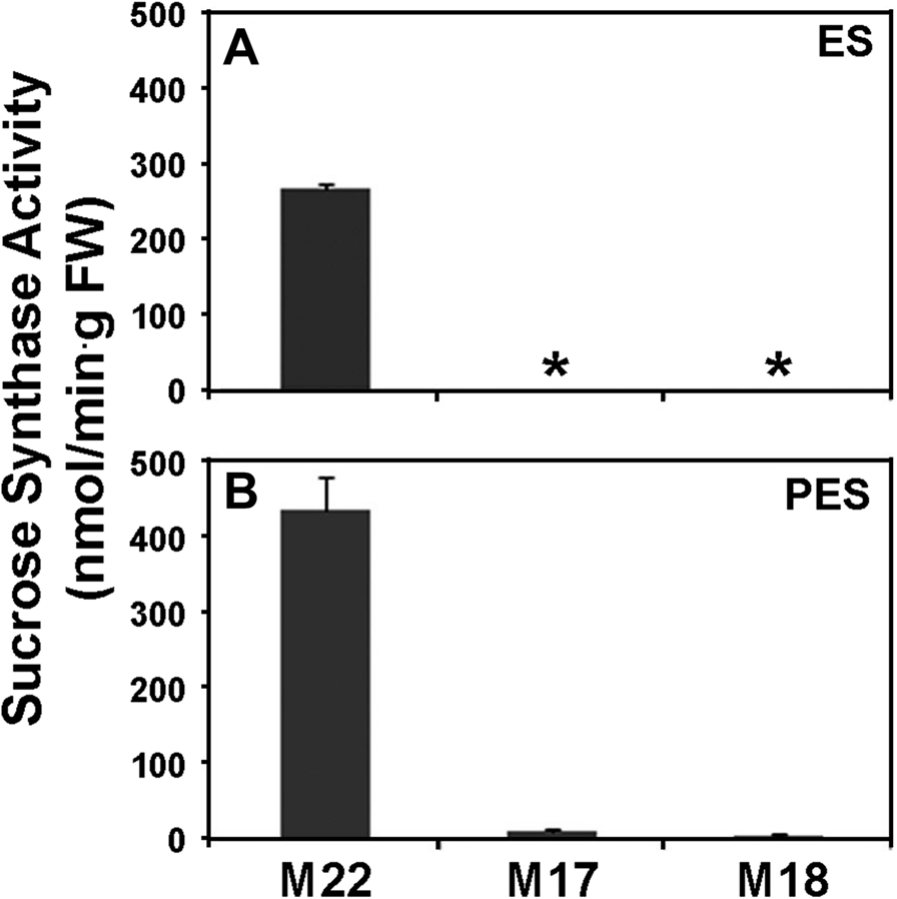
Sucrose synthase activity in stem internodes. Activity was measured in internodes of control (M22) and the lines containing the *PEPC7-P4::MsSUS1* construct (M17, M18). **a** elongating stem (ES) internodes. **b** post-elongating stem (PES) internodes. Values represent means ± standard error (*n* = 3). Asterisk indicates SUS activity was at the limits of detection

**Fig. 4:**
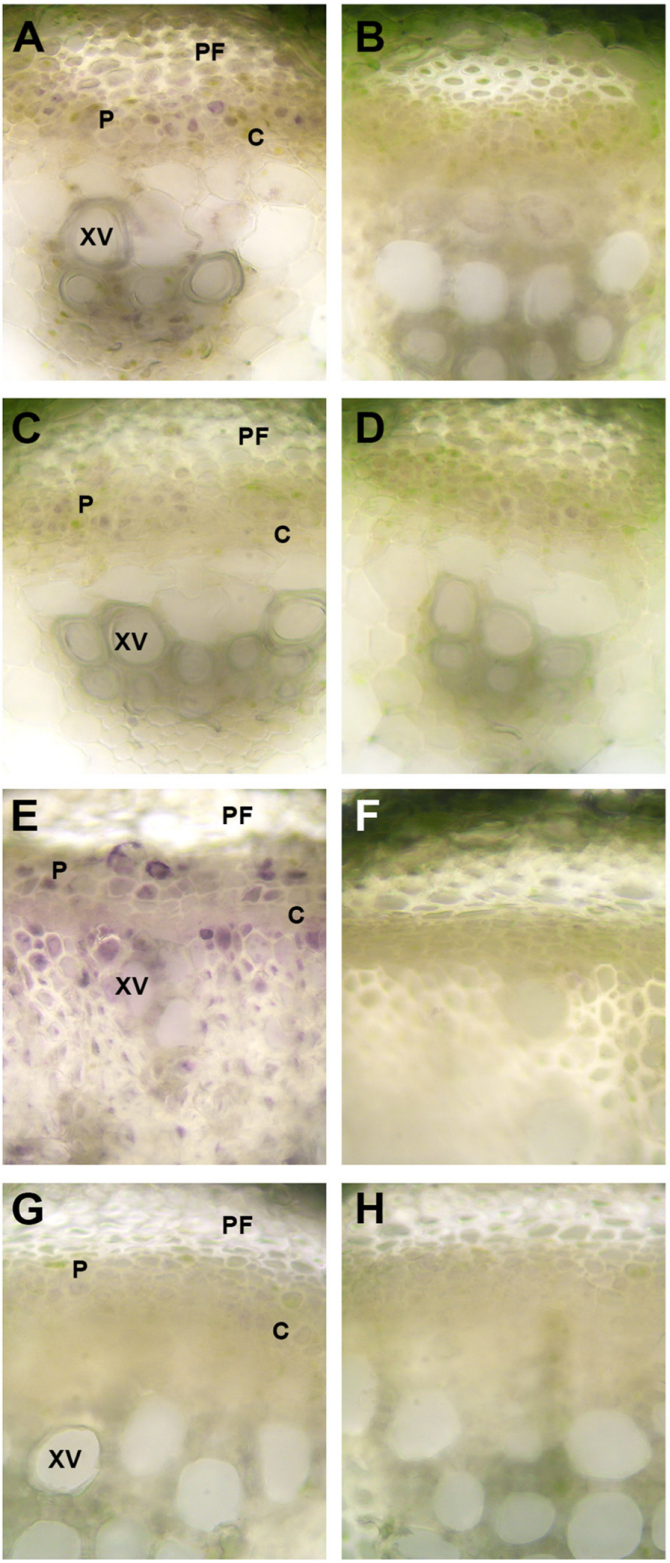
Comparison of *in situ* sucrose synthase activity in stem transverse-sections of elongating stem (ES) and post-elongating stem (PES) internodes of control (M22) and the SUS down-regulated (M18) transformant. Purple coloration in cells indicates enzyme activity. **a** ES internodes of M22; (**c**), ES internodes of M18; (**e**), PES internode of M22; (**g**), PES internode of M18. **b**, **d**, **f**, **h** are negative controls (no sucrose in assay medium) for **a**, **c**, **e**, **g**, respectively. Abbreviations: PF, phloem fibers; P, phloem; C, cambium; XV, xylem vessel

In stems of the control (M22) alfalfa line, SUS enzyme activity was found to be 1.6-fold higher in PES compared to ES internodes (Fig. 3). In the *PEPC7-P4::SUS1* transformed lines (M17, M18) SUS activity in ES internodes was below the level of detection (Fig. 3a) and SUS activity was reduced by more than 95 % in PES internodes compared to the control line M22 (Fig. 3b). SUS activity was detected by *in situ* enzyme assays in the phloem and xylem tissue in the control line with greater activity in the PES internodes than ES internodes (Fig. 4a, e). The *in situ* enzyme assays showed that SUS activity was greatly reduced in ES internodes of the *PEPC7-P4::MsSUS1* transformant (Fig. 4c) and was below the level of detection in PES internodes (Fig. 4g).

### SUS immunoblotting and mass spectrometry of SUS polypeptides

**Fig. 5:**
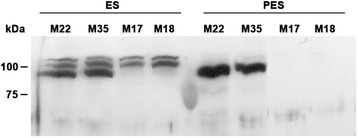
Immunoblot of sucrose synthase proteins in elongating stem (ES) and post-elongating stem (PES) internodes of control lines (M22, M35) and the lines containing the *PEPC7-P4::MsSUS1* construct (M17, M18). Each lane contains 40 μg of soluble protein from ES or PES internodes. Numbers at the side of the blot indicate the molecular mass of the protein markers in kDa

Immunoblotting was conducted to examine SUS protein in the soluble fraction (16,000 x g supernatant fraction) of stem extracts from the controls (M22, M35) and the *MsSUS1* down-regulated transformants (M17, M18). SUS antiserum produced against maize sucrose synthase 2 [21] was used to probe the immunoblot. The results showed that three immunoreactive polypeptides were detected in ES internodes in the control lines, one major polypeptide of approximately 90 kDa and two minor polypeptides of slightly higher molecular weight (Fig. 5). In the ES internodes of the SUS down-regulated transformed lines the major 90 kDa SUS polypeptide band was absent, although the two minor bands of higher molecular weight remained. In contrast, the PES internodes of control lines showed the presence of only one major SUS polypeptide band at approximately 90 kDa. The PES internodes of the SUS down-regulated lines showed no immunoreactive polypeptides.

**Fig. 6:**
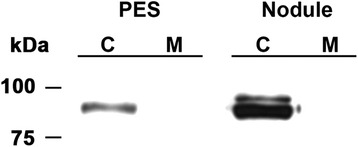
Immunoblot of sucrose synthase proteins in the cytosolic (C) and membrane (M) fractions of post-elongation stem (PES) internodes and nodules of control plants. Each lane contains 7.5 μg soluble protein. The protein in the membrane fraction was solubilized with 1 % Triton-X 100. Numbers at the side of the blot indicate the molecular mass of the protein markers in kDa

SUS protein can occur as cytosolic or membrane-associated [22–24]. The soluble fraction (16,000 x g supernatant) used in the immunoblot of PES internodes (Fig. 5) contained both the cytosolic and microsomal membrane fractions. To determine whether the SUS protein in PES internodes was cytosolic or membrane-associated, we centrifuged the soluble fraction at 100,000 x g to remove microsomal membranes and repeated the immunoblot. The results indicated that the SUS isoform in PES internodes was a soluble cytosolic protein and not a membrane-associated protein (Fig. 6). The immunoreactive band from the gel was eluted, trypsin digested, and analyzed by mass spectrometry. A total of 44 unique peptides were analyzed that corresponded to 426 of the 805 amino acids in SUS1 (53 % coverage). Based on available sequences in GenBank and RNA-seq data (http://plantgrn.noble.org/AGED/), the protein in the 90 kDa band was identified as MsSUS1.

One major and one minor immunoreactive SUS polypeptide were identified in an alfalfa root nodule extract from control plants (Fig. 6). Both SUS polypeptides in nodules were in the cytosolic fraction and no SUS protein was detected in the membrane-associated fraction. The immunoblot showed that the major SUS polypeptide in nodules co-migrates with the cytosolic MsSUS1 from stems. Mass spectrometry analysis of the major SUS polypeptide from nodules was also identified as MsSUS1. These results are consistent with the report that the *SUS1* orthologs in *M. truncatula* [11] and *L. japonicus* [12] are expressed in both nodules and stems.

### Effect of *MsSUS1* down-regulation on other *SUS* transcripts

**Fig. 7:**
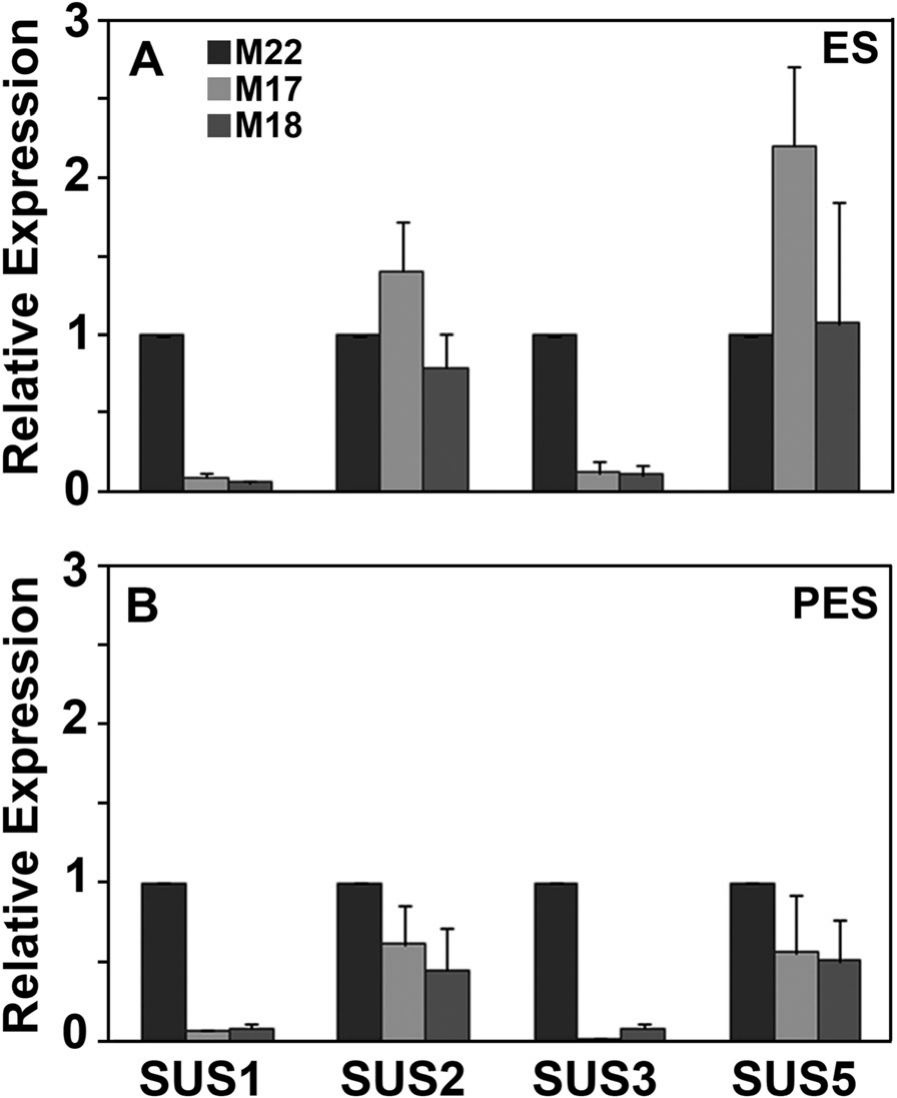
Quantitative reverse transcriptase-PCR analysis of transcripts for *MsSUS1, MsSUS2, MsSUS3*, and *MsSUS5* isoforms. **a** Elongating stem (ES) and (**b**) post-elongation stem (PES) internodes of a control line (M22) and lines containing the *PEPC7-P4::MsSUS1* construct (M17, M18). Values represent means ± standard error (*n* = 3)

We identified four *SUS* isoforms from alfalfa, *MsSUS1, MsSUS2, MsSUS3*, and *MsSUS5* (Additional file 1), using alfalfa RNA-seq data (http://plantgrn.noble.org/AGED/). The alfalfa genes identified are orthologs of the *MtSUS1*, *MtSUS2, MtSUS3*, and *MtSUS5* genes [25] previously identified in *M. truncatula* (Additional file 2)*.* Using the sequence data for the four alfalfa *SUS* isoforms, we designed primers for *MsSUS1, MsSUS2, MsSUS3*, and *MsSUS5* (Additional file 3) and measured transcript abundance of these four *SUS* isoforms in plants containing the *PEPC7-P4::MsSUS1* construct (M17, M18) relative to the transgenic control line M22. In both ES and PES internodes, *MsSUS3* transcripts had very low relative expression in the *PEPC7-P4::MsSUS1* transformed lines (Fig. 7) suggesting that the transgene also caused down-regulation of *MsSUS3.* However, down-regulation was not observed for *MsSUS2* and *MsSUS5* transcripts in the *PEPC7-P4::MsSUS1* transformed lines (Fig. 7). Expression of *MsSUS2* and *MsSUS5* in transformed lines is consistent with the presence of minor bands in the immunoblot of ES extracts (Fig. 5) and low levels of *in situ* SUS activity in ES in ternodes of *PEPC7-P4::MsSUS1* transformed lines (Fig. 4).

### SUS down-regulation does not yield a mutant phenotype

Although SUS enzyme activity was largely absent in stem internodes of M18 and M17 (Fig. 3), these plants did not exhibit an obvious shoot phenotype compared to the control (M22). We compared shoot and root biomass accumulation of the control (M22) and the *MsSUS1* down-regulated lines (Table 1). The results show a small (11 %) significant (*P* < 0.05) reduction in shoot and total biomass of the M17 line compared to the control (M22). However, no significant differences were observed between the M18 and M22 lines. We also examined the effects of *MsSUS1* down-regulation on stem cell wall sugar composition (Additional file 4). The results showed that the amount of cellulose (glucose) did not differ between the *MsSUS1* down-regulated lines (M17, M18) and the control (M22). Small statistically significant (*P* < 0.05) changes in total cell wall and galactose content occurred in M18 but not M17 (Additional file 4). It is possible that changes in cell wall content were localized to the vascular tissues and therefore significant differences could not be detected in the whole stem analysis.

**Table 1:** Dry weight (g) of shoots and roots of the control (M22) and the *MsSUS1* down-regulated lines (M17, M18)

Line	Shoot	Root	Total	R/S Ratio^a^
M22	4.80 (0.21)	1.83 (0.13)	6.63 (0.31)	0.38
M17	*4.26 (0.20)	1.62 (0.16)	*5.88 (0.33)	0.38
M18	4.41 (0.20)	1.65 (0.13)	6.06 (0.30)	0.37

Values represent means ± standard error (in parentheses), *n* = 15. Significant differences (*P* < 0.05) as determined by analysis of variance between M22 (control) and M17 are indicated by an asterisk. ^a^root/shoot ratio

### Effect of *MsSUS1* down-regulation on invertase and *in situ* enzyme assays of neutral invertase

**Fig. 8:**
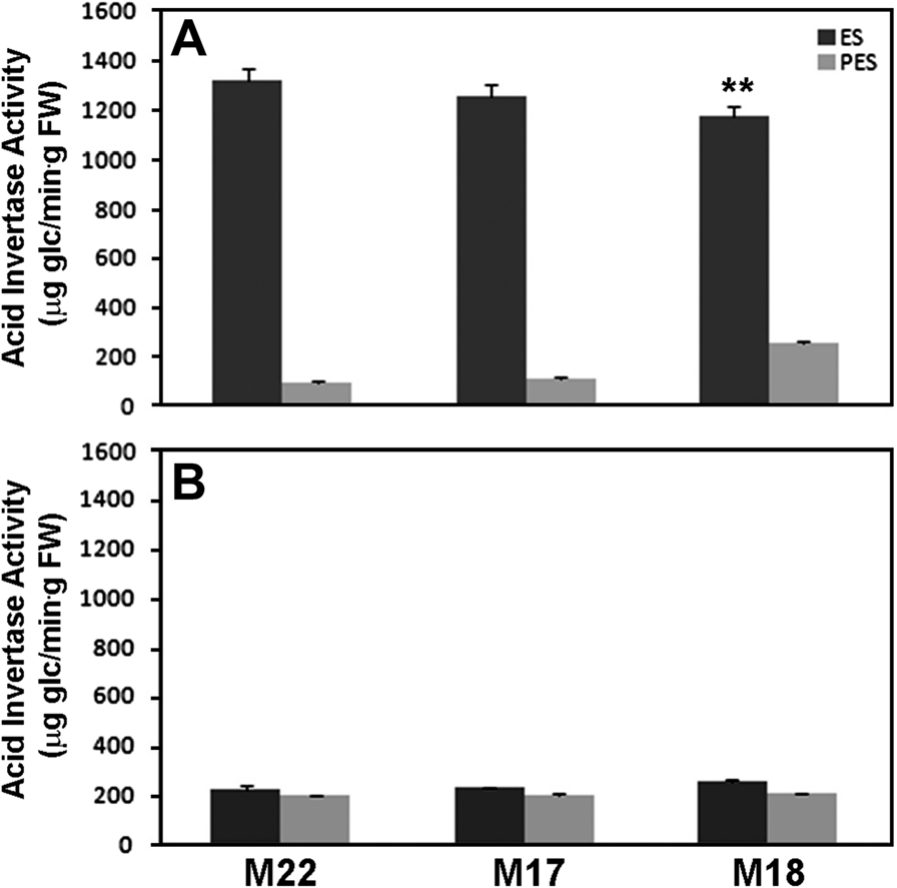
Acid invertase activity in elongating stem (ES) and post-elongation stem (PES) internodes. **a** Vacuolar and (**b**) cell wall activity of M22 (control) and the *MsSUS1* down-regulated lines (M17, M18). Values are means ± standard error (*n* = 3). ** indicates a significant difference (*P* < 0.01) as determined by analysis of variance compared to the control M22

We examined the possibility that the lack of a pronounced mutant phenotype in plants exhibiting significant *MsSUS1* down-regulation in stems was the result of invertase activity. Therefore, we measured acid, alkaline, and neutral invertase activity in ES and PES internodes of the control and down-regulated lines. For acid invertase we evaluated the activity of vacuolar (soluble) and cell wall (insoluble) forms in ES and PES internodes. The results showed that vacuolar acid invertase activity was very high in ES relative to PES internodes (Fig. 8a) in both the control (M22) and *MsSUS1* down-regulated lines (M17, M18). This result was expected because vacuolar acid invertase plays a role in osmoregulation and is highly expressed in regions of cell division and elongation [26], which occur in ES internodes. Vacuolar acid invertase was slightly reduced in ES internodes of M18 compared to the control M22 but was not significantly different in ES internodes of M17 compared to the control (Fig. 8a). There were no significant differences in vacuolar acid invertase activity between control and down-regulated lines in PES internodes. Insoluble acid invertase activity showed no significant differences between the ES and PES samples and was expressed at a relatively low level as compared to the vacuolar acid invertase (Fig. 8b). *MsSUS1* down-regulated lines showed no significant difference in insoluble acid invertase activity compared to the control. Overall, the results indicated that *SUS1* down-regulation had little or no effect on acid invertase activity in either ES or PES internodes.

**Fig. 9:**
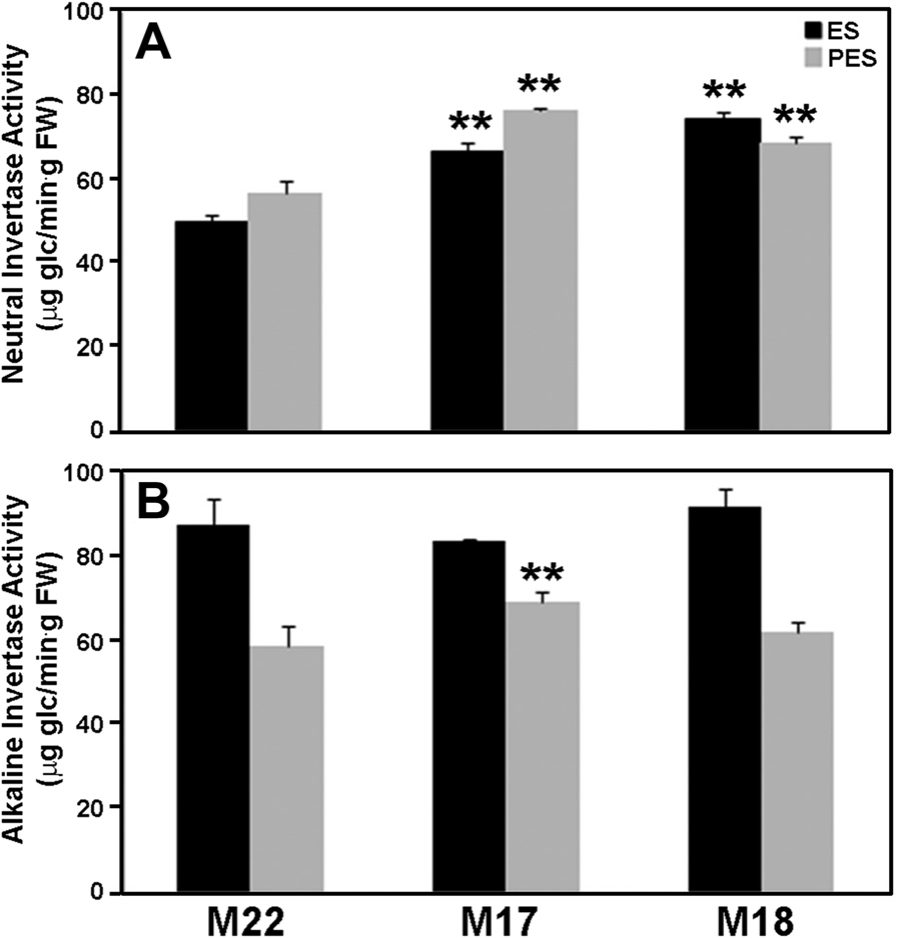
Comparison of invertase activity in elongating stem (ES) and post-elongation stem (PES) internodes. **a** Neutral and (**b**) alkaline invertase activity of the control (M22) and *MsSUS1* down-regulated lines (M17, M18). Values represent the mean ± standard error (*n* = 3). ** indicates a significant difference (*P* < 0.01) as determined by analysis of variance compared to the control M22

**Fig. 10:**
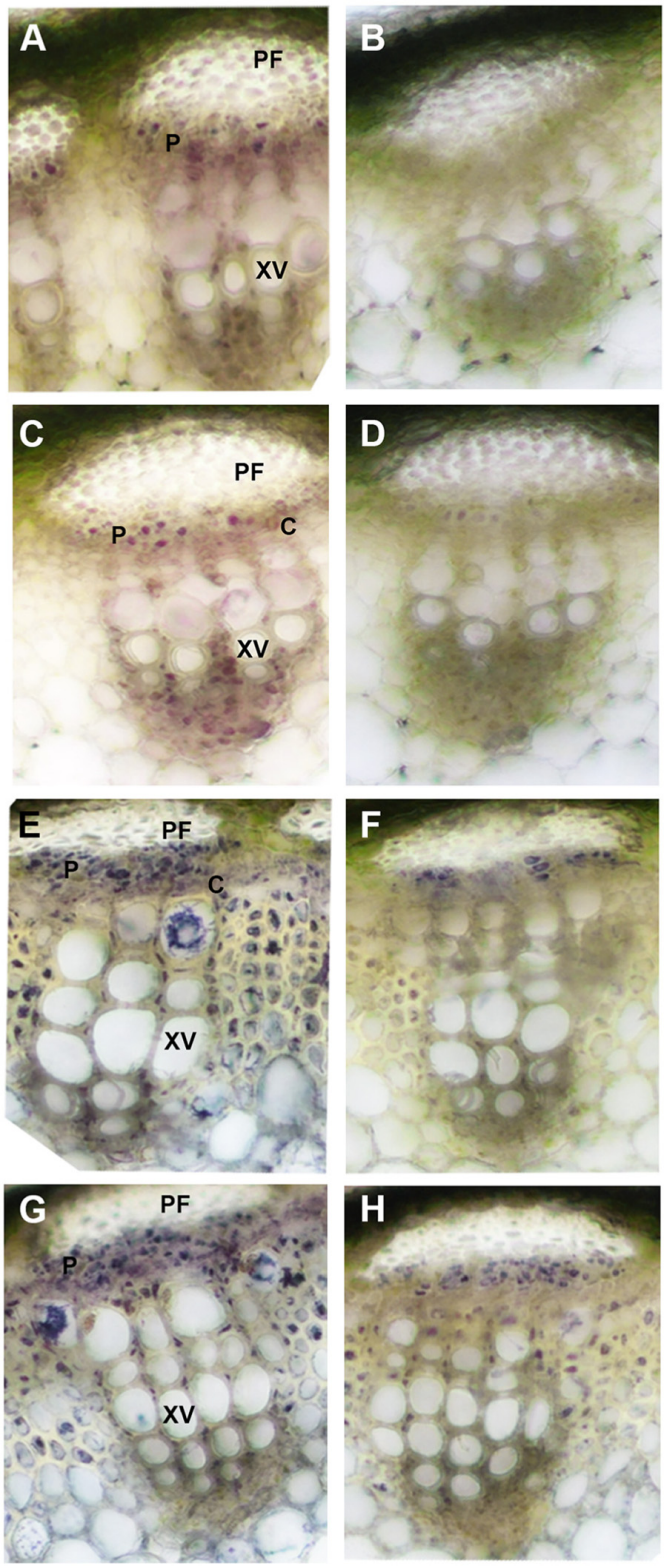
Comparison of *in situ* neutral invertase activity measured in stem transverse-sections of elongating stem (ES) and post-elongation stem (PES) internodes of control (M22) and the *MsSUS1* down-regulated line (M18). **a** ES internode of M22; (**c**), ES internode of M18; (**e**), PES internode of M22; (**g**), PES internode of M18. **b**, **d**, **f**, **h** are negative controls (no sucrose in the assay medium) for **a**, **c**, **e**, **g**, respectively. Abbreviations: PF, phloem fiber; P, phloem; C, cambium; XV, xylem vessel

In contrast to acid invertase activity, *MsSUS1* down-regulation resulted in significant (*P* < 0.01) increases in neutral invertase activity. Neutral invertase activity increased 1.3- to 1.5-fold in ES internodes of M17 and M18, respectively compared to M22 (Fig. 9a). Similarly, in PES internodes neutral invertase increased 1.4- and 1.2-fold in M17 and M18, respectively compared to M22. In contrast, there was no significant difference in alkaline invertase activity in ES internodes between control and *MsSUS1* down-regulated lines (Fig. 9b). Alkaline invertase activity in PES internodes of M17 was 1.2-fold higher than the control M22 but activity in M18 was similar to the control. An *in situ* enzyme assay of ES and PES stem transverse sections was done to localize neutral invertase activity in control and *MsSUS1* down-regulated lines. The results showed that neutral invertase activity was localized to the same vascular tissues (xylem, phloem) as SUS (Fig. 10a, e). Additionally, neutral invertase activity was maintained in vascular tissue of the M18 down-regulated transformant where SUS activity was greatly reduced (Fig. 10c, g).

## Discussion

This study investigated the effects of down-regulation of *MsSUS1* in ES and PES internodes of alfalfa. Our original objective, to over-express *MsSUS1* in alfalfa stem vascular tissue in order to increase cellulose in stem vascular tissues, resulted instead in down-regulation of *MsSUS1* transcripts (Fig. 2) and SUS enzyme activity (Fig. 3). The down-regulation of SUS in alfalfa stem tissue resulted in no obvious phenotype and no significant changes in cell wall sugar composition. However, A/N invertase activity was found to increase in stems of the SUS down-regulated lines and acid invertase levels remained high. Previous studies in *A. thaliana* and *L. japonicus* found that loss of SUS activity causes little to no change in plant phenotype whereas loss of invertase activity results in severe growth retardation [12, 17, 18]. Our results suggest that MsSUS1 and MsSUS3 are not necessary for normal vegetative growth in alfalfa and support a major role for invertase in sucrose catabolism. However, we cannot rule out that A/N invertase and possibly acid invertase in vascular tissue can compensate for reduced SUS activity.

Down-regulation of *SUS1* transcripts in stems of plants containing the *PEPC7-P4::MsSUS1* construct may be due to transgene silencing (co-suppression). Transgene silencing occurs when over-expression of a transgene results in down-regulation of both the transgene and endogenous homologous gene [27–29]. Our results show that *MsSUS1* is highly expressed in vascular tissue of alfalfa stems. The PEPC7-P4 promoter used for expression of coding sequences was shown to result in high levels of expression of GUS in vascular tissue (Fig. 1). In the transformed alfalfa plants generated in this study, expression of *MsSUS1* in stem vascular tissues, where it was already highly expressed, likely resulted in silencing. Results from qRT-PCR assays indicate that expression of *PEPC7-P4::MsSUS1* may also have silenced *MsSUS3* (Fig. 7). A previous study, in which over-expression of SUS increased cellulose in hybrid poplar, utilized a heterologous *SUS* gene from cotton and heterologous promoters [15]. The mechanism of co-suppression is not completely understood but is thought to occur when transcripts exceed a specific threshold [30]. Apparently, transgene expression of *MsSUS1* in vascular tissue exceeded the threshold required to trigger co-suppression. We also found *MsSUS1* expression to be down-regulated in roots and nodules of plants containing the *PEPC7-P4::MsSUS1* construct (unpublished results) indicating that co-suppression occurred throughout the plants.

Previous research in *A. thaliana*, *M. truncatula*, and *L. japonicus* identified multiple isoforms of *SUS* that are expressed with some organ specificity [8, 11, 12]. In *M. truncatula*, *MtSUS1* is the predominantly expressed isoform in all organs assayed and expression is enhanced in vascular cells of the stem, root, and nodule [25]. The isoform *MtSUS3* is also highly expressed in stems of *M. truncatula*. To clarify the roles of the six *SUS* isoforms in *A. thaliana*, single and double knockout mutants were constructed. Elimination of specific isoforms did not result in an obvious phenotype. However, a *sus1/sus4* mutant had reduced weight gain under hypoxic soil conditions [8]. Additional studies with a quadruple mutant (*sus1/sus2/sus3/sus4*) found no change in starch and sugar content in leaves or roots, seed weight or lipid content, cellulose content, or cell wall structure compared to the wild type [17]. In contrast, the *A. thaliana* double mutant of cytoplasmic neutral invertase (*cinv1/cinv2*) had reduced root and shoot growth as well as abnormally large cells in the root expansion zone, and abnormal cell division in the stele, endoderm, and cortex indicating a critical role of invertases for normal growth [17]. Studies in *L. japonicus* found that the predominant invertase isoform, LjINV1, is crucial to whole plant development but is not essential for nodule formation or function [18]. Sucrolytic activity is required in vascular tissue for energy metabolism, synthesis of structural carbon for callose and cell wall cellulose synthesis, and to maintain turgor for the proper functioning of the transport stream in the phloem [16, 26, 31]. Our results indicate that invertases can supply the sucrolytic activity needed in vascular tissue. However, we cannot rule out compensation of sucrolytic activity by additional SUS isoforms. The four alfalfa *SUS* isoforms that we identified could be placed into two groups depending on their pattern of expression relative to the control (Fig. 7). *MsSUS1* and *MsSUS3* had very low transcript accumulation in the down-regulated lines relative to the control. In contrast, *MsSUS2* and *MsSUS5* transcript levels were similar to or slightly higher in the *MsSUS1* down-regulated lines relative to the control. Minor bands identified in the immunoblot of stem internode extracts (Fig. 5) may correspond to these isoforms. The minor bands are likely not MsSUS1 because those bands are lacking in the down-regulated plants. We sequenced similar minor bands from gels separating proteins extracted from roots of the same transgenic lines and identified MsSUS2 and MsSUS3 (unpublished results).

Previous reports on the essential function of invertase in nonphotosynthetic organs [17, 18, 32, 33] highlights the need for additional research on the regulation and function of invertases in higher plants including their role in signaling pathways regulating carbon exchange and starch accumulation. The presence of A/N invertase in multiple cellular locations (cytosol, chloroplasts, mitochondria, nuclei) suggests a role in coordinating metabolic processes within and between organelles. Cytoplasmic invertases have been postulated to play a key role in maintaining sugar homeostasis in cells where SUS activity is low by controlling cytosolic concentrations of sucrose, glucose and fructose [34]. Our study showed that acid invertase activity was high and A/N invertases were elevated in both ES and PES internodes in the *MsSUS1* down-regulated plant lines relative to the controls (Fig. 9). In PES internodes, in which secondary cell wall synthesis was occurring, A/N invertase activity was 1.2- to 1.4-fold higher in *MsSUS1* down-regulated lines than in PES internodes of the control. This suggests that invertase was supplying the glucose required for cellulose synthesis. Invertase may also be supplying the sucrolytic activity needed for maintenance of sucrose translocation in phloem. In stems there is a constant leak of sucrose from transport phloem because of the large concentration gradient between phloem and the apoplast [35]. It is critical that the leaked sucrose be retrieved to maintain the turgor pressure that drives the flow of sucrose from source to sink. The sucrose retrieval mechanism involves uptake via a sucrose/proton symporter that utilizes the proton motive force generated by the plasma membrane H^+^-ATPase [36]. Sucrose cleavage provides the carbon needed to generate ATP to fuel the plasma membrane H^+^-ATPase. If the sucrose retrieval function in phloem depended on sucrose cleavage by SUS, significant reductions in sucrose translocation would be expected in the *MsSUS1* down-regulated lines resulting in growth inhibition. Because SUS down-regulation in vascular tissue caused only a small reduction in plant biomass accumulation, it appears that invertases activity in the phloem is able to provide the energy needed for sucrose retrieval, but there may be an energetic penalty. Sucrose cleavage by invertase is less energy efficient than cleavage by SUS. It is also possible that alfalfa stems experience hypoxia (oxygen deficiency) due to limited oxygen diffusion and high rates of metabolism. Hypoxia causes a switch from aerobic to anaerobic respiration, which is less efficient for ATP production. In hypoxic conditions, sucrose cleavage by SUS would be energetically advantageous over hydrolysis by invertase. In *MsSUS1* down-regulated plants the energy penalty of sucrose cleavage by invertase might be expected to result in a slower growth rate and/or lower biomass accumulation. The small significant reduction in shoot biomass accumulation observed in down-regulated plants compared to the control suggests that alfalfa stems may experience hypoxia and the invertase activity in *MsSUS1* down-regulated plants has an energetic penalty.

SUS has been reported to have additional important roles in plant growth and development. In legumes, root nodules contain high levels of SUS [12, 25] and it has been suggested that pericycle cells in the nodule vascular system play a key role in sucrose transport into the nitrogen-fixing region and the loading of nitrogenous compounds produced by nitrogen fixation into the xylem [37]. Additionally, most research indicates that SUS activity is enhanced while A/N invertase activity is reduced in roots exposed to anoxia or hypoxia [14, 38, 39]. The SUS down-regulated alfalfa lines can be useful tools for future investigations into the relative roles of SUS and invertase in sucrose transport and osmotic stress tolerance.

## Conclusions

We examined if expression of *MsSUS1* in vascular tissue would increase cellulose content of alfalfa cell walls. In contrast to expectations, alfalfa plants transformed with an alfalfa *MsSUS1* cDNA using an alfalfa promoter for vascular-specific expression resulted in down-regulation of transcripts, protein, and SUS enzyme activity in stem internodes. Down-regulation was most likely due to transgene silencing (co-suppression). However, down-regulation of SUS activity had only minor effects on plant dry weight or cell wall content of stems. A/N invertase activity increased in vascular cells of *MsSUS1* down-regulated plants and invertase appeared to provide the sucrolytic activity required for cell wall synthesis and for maintenance of sucrose translocation in phloem.

## Methods

### Construction of plant transformation vectors

Previously, the promoter fragment PEPC7-P4 of the alfalfa nodule-enhanced phosphoenolpyruvate carboxylase gene *PEPC-7* (L39371) consisting of nucleotides −592 to 86 relative to the transcription start site was cloned and inserted into the *Xba*I and *Sma*I restriction sites in the plant expression vector pBI101.1 [40] producing the *PEPC7-P4::GUS* chimeric reporter gene [19]. Sequencing the PEPC7-P4 promoter during this study revealed a 56 bp direct repeat that was not reported in the original gene sequence submission. A sequence correction has been submitted to GenBank for the *PEPC-7* gene. In an earlier study, the cDNA from a nodule-enhanced sucrose synthase gene (*MsSUS1*; AF049487) was isolated and cloned into pBluescript [41]. For this study, the *gusA* sequence in the *PEPC7-P4::GUS* construct was replaced with an *Xma*I-*Sac*I fragment containing the *MsSUS1* cDNA to produce the *PEPC7-P4:: MsSUS1* expression vector. The nucleotide sequences of the cloned DNA fragments were verified by sequencing at the University of Minnesota BioMedical Genomics Center.

### Plant transformation and selection of transformed lines

Alfalfa (cultivar Regen SY) was transformed with the *PEPC7-P4::GUS* or the *PEPC7-P4:: MsSUS1* construct by *Agrobacterium tumefaciens*-mediated transfer as previously described [42]. Transformed plants were selected by kanamycin resistance and confirmed to be transgenic by the presence of *nptII* by PCR assays as described previously [43]. The transformants selected to be used as transgenic control plants (M22, M35) contained the *PEPC7-P4::GUS* construct but lacked GUS expression as tested by histochemical staining. The transformants containing the *PEPC7-P4:: MsSUS1* construct selected for further evaluation (M17, M18) had the lowest *MsSUS1* expression as measured by qRT-PCR. The presence of the *PEPC7-P4:: MsSUS1* construct in *MsSUS1* down-regulated plants was confirmed by PCR using primers in the promoter and *MsSUS1* coding sequence.

### Plant material and culture conditions

Selected primary transformants were propagated clonally by stem cuttings and grown in the greenhouse. Primary transformants were used due to the severe inbreeding depression in alfalfa. For most experiments, plants were grown in a sand:soil mixture (2:1,v/v), one plant per cone-tainer (Stuewe & Sons, Tangent, OR; 7 cm width, 35 cm depth). Plants were grown in a randomized complete block with three or four replicates. Plants were watered weekly with quarter-strength Hoagland’s nutrient solution containing 25 ppm N [44]. For qRT-PCR, immunbloting, and enzyme assay experiments, 10 stems were harvested from each replicate. ES internodes (apical four to five internodes) and PES internodes (seventh or eighth internode from the stem apex) were harvested from flowering plants as previously described [45]. Stem material from each replicate (approximately 1 g fresh weight) was combined, frozen in liquid nitrogen, and stored at −80 °C until assayed. For experiments comparing growth of transformants, plants were grown in lime-amended sand and were watered with half-strength Hoagland’s nutrient solution containing 100 ppm N. Every fourth day, plants received only water. Plants were harvested at the time of flowering. Dry weight of roots and shoots were measured after drying at 60 °C. For examining expression of *PEPC7-P4::GUS* in roots and nodules, plants were grown in quartz sand. Plants were inoculated with *Sinorhizobium meliloti* (Nitragin®, Novozymes, Davis, CA) and watered daily with half-strength Hoagland’s nutrient solution without N.

### Quantitative reverse-transcriptase PCR (qRT-PCR)

RNA was isolated from ES and PES samples using the RNeasy Plant Mini kit (Qiagen, Valencia, CA). Following DNase I treatment with the DNA-free kit (Ambion Inc., Austin, TX), first strand cDNA for each sample was made from 2 μg total RNA using Superscript II RT (Invitrogen, Carlsbad, CA) following the manufacturer’s recommendations and diluted 10-fold before use in PCR. Gene-specific primers (Additional file 3) were designed based on *MsSUS* isoform sequences retrieved from GenBank and alfalfa RNA-seq data (Additional file 1; http://plantgrn.noble.org/AGED/). qRT-PCR was performed using the iTaq Universal SYBR Green Supermix (BioRad, Hercules, CA) in 12.5 μL reactions containing 4 pmol of each forward and reverse primer and 2.5 or 3.0 μL of template cDNA. Samples from three biological replicates were run in triplicate on a StepOnePlus^TM^ Real-Time PCR System (Applied Biosystems, Grand Island, NY) following the manufacturer’s recommendations. The PCR conditions were as follows: 30 s of pre-denaturation at 95 °C, 40 cycles of 3 s at 95 °C and 30 s at 60 °C, followed by steps for melting curve generation (15 s at 95 °C, 60 °C, 95 °C). The StepOne software (Applied Biosystems) was used for data collection. Disassociation curves for each amplicon were examined to confirm presence of a single amplicon. Melting curves showed that only one *SUS* transcript was measured demonstrating that the primers were specific for transcripts of each isoform. Relative transcript accumulation for each sample was obtained using the comparative C_t_ method [46] using the C_t_ value of the alfalfa actin gene (JQ028730.1) for sample normalization.

### SUS enzyme assay

ES and PES internode samples (0.3 g) were ground with a mortar and pestle in 3.0 mL extraction buffer [100 mM MES, pH 6.8, 15 % (v/v) ethylene glycol, 2 % (v/v) β-mercaptoethanol, 60 mg polyvinylpolypyrrolidone (PVPP), 30 μL of 0.1 M phenylmethanesulfonylfluoride (PMSF), and 30 μL protease inhibitor cocktail (Sigma-Aldrich, St. Louis, MO)]. The homogenate was centrifuged (16,800 x g, 25 min) at 4 °C. The supernatant was applied to a desalting column (PD minitrap G-25, GE Healthcare, Buckinghamshire, UK) that had been equilibrated in extraction buffer. The eluent was used for the assays. Enzyme activity was assayed in a 1 mL reaction mixture containing 50 mM HEPES, pH 7.4, 2 mM magnesium acetate, 5 mM dithiothreitol (DTT), 2 mM EGTA, 50 mM sucrose, 1 mM potassium pyrophosphate, 1 mM UDP, 1 mM NAD, 0.02 mM D-glucose-1,6-diphosphate, and 1 unit each of phosphoglucomutase, uridine-5′-diphosphoglucose pyrophosphorylase, and glucose 6-phosphate dehydrogenase (from *Leuconostoc mesenteroides*; Sigma-Aldrich). Enzyme activity was monitored by measuring absorbance at 340 nm (24 °C) using a Thermo Scientific Genesys 6 spectrophotometer (Thermo Electron Corp., Madison, WI).

### Invertase enzyme assays

The acid invertase assay procedure was adapted from Sergeeva et al. [47]. Frozen tissue (0.4 g) from ES and PES internodes was ground in a mortar and pestle with liquid nitrogen then homogenized in 2 mL extraction buffer [50 mM HEPES · KOH, pH 7.4, 5 mM MgCl_2_, 1 mM EGTA, 1 mM EDTA, 5 mM DTT, 10 % glycerol, 40 mg PVPP, 20 μL 0.1 M PMSF, 20 μL protease inhibitor cocktail for plant cell and tissue extracts (Sigma-Aldrich)]. The homogenate was centrifuged (16,800 x g, 1 min, 4 °C) and the supernatant was transferred to a fresh tube on ice for assay of soluble (vacuolar) acid invertase. The pellet was washed three times with 1 mL of extraction buffer minus PVPP and DTT. Washing involved homogenizing the pellet followed by centrifugation (16,800 x g, 4 °C). Centrifugation lasted 1 min for the first two washes and 5 min for the final wash. The washed pellet was resuspended in extraction buffer consisting of 20 mM MES · KOH, pH 6.0, 1 M NaCl and incubated overnight at 4 °C. The next day the suspension was centrifuged (16,800 x g, 20 min, 4 °C) and the supernatant was transferred to a fresh tube on ice for assay of the cell wall bound acid invertase. Both cell wall and soluble acid invertase were measured using a two-step assay involving glucose formation (reaction A) and subsequent spectrophotometric assay of glucose involving NAD reduction (reaction B). For assay of soluble acid invertase, the reaction A medium contained 150 μL plant extract, 200 μL of 150 mM citrate-phosphate buffer, pH 4.5, and 50 μL of 400 mM sucrose. The assay was conducted at 30 °C and stopped after 10 min by placing tubes in a boiling water bath for 4 min. The tubes were centrifuged at room temperature to pellet precipitated proteins. Controls were run without added sucrose and used for background subtraction in the final calculations. The reaction B assay cuvette contained 450 μl of 2X HEPES assay buffer (100 mM HEPES, pH 7.4, 4 mM Mg acetate, 10 mM DTT, 4 mM EGTA), 100 μL substrate mix (10 mM NAD and 5 mM ATP in sterile water) and boiled reaction A (25 μL and 100 μL for ES and PES extracts, respectively) and water (375 μL and 300 μL for ES or PES tissue extracts, respectively). The reaction was run to completion (3 min) and background absorbance was measured at 340 nm using a Thermo Scientific Genesys 6 spectrophotometer. Next, 50 μL of an enzyme mix [1 unit each of hexokinase and glucose 6-phosphate dehydrogenase (from *L. mesenteroides*; Sigma-Aldrich) in 2x HEPES assay buffer] was mixed into each assay cuvette and incubated for 15 min at room temperature. Absorbance readings were taken on each reaction and the change in absorbance was used to calculate glucose content. For assay of cell wall acid invertase, the assay protocol was the same as for the soluble acid invertase assay except that 50 μL of cell wall extract was used for reaction A conducted at pH 4.8 and 200 μl of reaction A was used for the NAD reduction assay (reaction B).

For alkaline and neutral invertase assays, tissue was extracted in the buffer used for extracting acid invertase except PVPP was not included. The extract was centrifuged (16,800 x g, 25 min, 4 °C). Activities of both enzymes were measured in a two-step assay as described above for acid invertase except that the buffers of reaction A consisted of 100 mM HEPES-NaOH (pH 7.5) or 100 mM sodium borate (pH 9.4) for measuring neutral and alkaline invertase activity, respectively. Reaction A contained either 100 μL ES or 50 μL PES extract for measuring neutral invertase and 100 μL ES or PES extract for measuring alkaline invertase. For the neutral invertase assay of ES and PES extracts, reaction B contained 200 μL boiled reaction A. For the assay of alkaline invertase, reaction B contained 200 μL or 100 μL boiled reaction A for ES and PES extracts, respectively. Alkaline and neutral invertase assays were also run in the presence of Tris to check for known Tris inhibition.

Assays were run in triplicate on three biological replicates. Statistical analyses were performed using the analysis of variance function of InStat (GraphPad, San Diego, CA) with Dunnett’s multiple comparison post-hoc test to compare down-regulated lines to the control line.

### Protein extraction and immunoblotting

Proteins were extracted by grinding 0.4 g stem tissue with a mortar and pestle in liquid nitrogen followed by the addition of 2 mL extraction buffer (100 mM MES, pH 6.8, 15 % ethylene glycol, 2 % β-mercaptoethanol, 100 mM sucrose, and 20 μL of 0.1 M PMSF). Following centrifugation (16,000 x g*,* 20 min, 4 °C) the supernatant was transferred to a fresh tube and the protein was quantified using the Bio-Rad Protein Assay reagent (Bio-Rad, Hercules, CA). Proteins were separated on a 10 % SDS-PAGE gel [48] and transferred to nitrocellulose. SUS was detected with an antiserum produced against maize sucrose synthase 2 (SS2; [21]). Precision Plus Dual Protein standards (Bio-Rad) were used to estimate molecular weight. Bound antibodies were visualized with the SuperSignal West Pico Chemiluminescent Substrate kit (Thermo Scientific, Rockford, IL).

### Mass spectrometry of SUS proteins

Total soluble proteins of PES internodes from M22 (control) were extracted as described above and separated on a 10 % SDS-PAGE gel. A protein band corresponding to the MsSUS1 antibody-reactive band, determined by immunoblot analysis of an identical lane run on the gel, was excised and an in-gel tryptic digest was performed. The identity of MsSUS1 from M22 PES tissue was confirmed by MS/MS performed by the University of Minnesota Center for Mass Spectrometry and Proteomics. LC/MS samples were analyzed using Sequest (Thermo Fisher Scientific, San Jose, CA, version 27). Sequest was set up to search the non-redundant *M. truncatula* database modified to include the GenBank entry AF049487 for the *MsSUS1* transcript. Scaffold (version 3.6.0, Proteome Software Inc., Portland, OR) was used to validate LC/MS based peptide and protein identifications. Peptide identifications were accepted if they were established at greater than 95 % probability as specified by the Peptide Prophet algorithm [49]. Protein identifications were accepted if they could be established at greater than 99 % probability and contained at least two identified peptides. Protein probabilities were assigned by the Peptide Prophet algorithm [50].

### Sucrose synthase and neutral invertase *in situ* enzyme assays

SUS and neutral invertase *in situ* enzyme assays were conducted by coupling enzyme activity to the reduction and precipitation of nitro-blue tetrazolium (NBT). Transverse sections of ES and PES samples (100–150 μm) were cut using a sledge microtome. The sections were immediately fixed by applying fixation medium to the knife and to each freshly prepared stem section. The fixation medium contained 1 % paraformaldehyde, 2 % (w/v) polyvinylpyrrolidone 40, and 5 mM DTT, pH 7.0. The tissue was fixed at room temperature for 30 min to 1.5 h. After fixation, sections were rinsed three times in water followed by a 3 h water wash at 4 °C. Cold water was refreshed once during the 3 h period. The sucrose synthase assay was performed as described by Wittich and Vreugdenhil [51] except that the concentration of sucrose was 100 mM and the incubation period was 1 h at room temperature. The assay medium contained: 100 mM sucrose, 15 mM UDP, 15 mM PPi, 150 mM NAD, 3 mM glucose-1,6-bisphosphate, 1 unit phosphoglucomutase, 1 unit glucose-6-phosphate dehydrogenase, 1 unit UDPG pyrophosphorylase, 50 mM HEPES-KOH, pH 7.5, 1 mM EDTA, 1 mM EGTA, 0.1 % bovine serum albumin (BSA), 5 mM MgCl_2_, and 0.07 % NBT. The neutral invertase *in situ* assay medium contained: 50 mM HEPES-KOH, pH 7.5, 1 mM EDTA, 1 mM EGTA, 1 mM NAD, 5 mM ATP, 100 mM sucrose, 0.1 % BSA, 0.03 % NBT, and 1 unit each of glucose-6-phosphate dehydrogenase, hexokinase, and phosphoglucoisomerase, 100 mM sucrose, 1 mM NAD, 5 mM ATP, and 0.03 % NBT. Assays were conducted at room temperature for 1 h for ES internodes and overnight for PES internodes. Negative controls were conducted without sucrose in the assay medium.

### Histochemical GUS analysis

GUS activity was detected as described by Udhe-Stone et al. [52]. Micrographs of stained stem sections were taken using a Nikon SMZ800 stereoscope attached to a Nikon DXM1200 digital camera.

### Cell wall analysis

Ten stems (45 cm measured from the apex) were harvested at flowering and combined for each replicate. Stem cell wall sugar composition was determined using the Uppsala dietary fiber method [53] as previously described [54]. Statistical analyses were performed using the analysis of variance function of R [55].

### Availability of supporting data

The data sets supporting the results of this article are included within the article and its additional files.

## Additional files

Additional file 1:
**DNA sequences used for qRT-PCR primer design.** (DOCX 166 kb) Additional file 2:
**Phylogenetic analysis of** ***SUS genes in alfalfa (Ms)***, ***Medicago truncatula (Mt)***, ***Arabidopsis thaliana (At)*** **and**, ***Pisum sativum (Ps).*** (DOCX 181 kb) Additional file 3:
**Primer sequences used for qPCR.** (DOCX 62 kb) Additional file 4:
**Cell wall content of control (M22) and the** ***PEPC7-P4::MsSUS*** **transformants (M17, M18).** (DOCX 64 kb)
