# Supplementary material for: Transgene silencing of sucrose synthase in alfalfa (Medicago sativa L.) stem vascular tissue suggests a role for invertase in cell wall cellulose synthesis
Source: BMC Plant Biol. 2015 Dec 1;15:283. doi: 10.1186/s12870-015-0649-4 (PMC4666122; doi:10.1186/s12870-015-0649-4)
Supplement: Additional file 1: — DNA sequences used for qRT-PCR primer design. (DOCX 166 kb) [file 12870_2015_649_MOESM1_ESM.docx]

**Additional file 1.** DNA sequences used for qRT-PCR primer design.

>MsSUS1_GenBank AF049487.1

TCCGCTTTACACCCCCCTTTCTTTTTTGCGTTCACTCTGTTTTCATAGTTGAAGATTTTCAATGGCTACAGAACGTTTGACTCGTGTTCATAGTCTCAAGGAGAGACTTGATGAAACCTTAACTGCTAATAGGAATGAAATTTTGGCCCTTCTTTCAAGGCTTGAAGCAAAGGGAAAGGGAATTTTGCAACACCATCAAGTGATTGCTGAGTTTGAGGAAATTCCTGAAGAGAGTAGACAGAAGCTGACTGATGGTGCATTTGGTGAAGTTTTGAGATCCACACAGGAAGCAATAGTTTTGCCACCATGGGTTGCACTTGCTGTTCGTCCAAGGCCAGGTATTTGGGAGTATCTGAGAGTGAATGTGCATGCTCTTGTTGTCGAAAATTTGCAACCTGCTGAGTTTCTCAAATTCAAAGAAGAACTTGTTGATGGAAGTGCTAATGGAAACTTTGTGCTTGAGTTGGACTTTGAACCATTTACTGCATCTTTCCCTCGTCCTACTCTCAACAAGTCAATTGGAAATGGTGTGCACTTCCTTAATCGCCATCTTTCTGCTAAACTCTTCCATGACAAGGAGAGTTTACATCCACTTTTGGAATTTCTCAGACTTCACAGCTACAAGGGAAAGACATTGATGTTGAATGACAGAATTCAAAACCCCGATTCTCTTCAACATGTTCTGAGGAAAGCTGAAGAGTATCTAAGCACAATTGATCCTGAAACACCATACTCAGAATTTGAACACAGGTTCCAGGAGATTGGTTTGG

AGAGAGGCTGGGGAGACACCGCAGAGCGCGTCCTCGAGTCTATCCAACTTCTCTTGGATCTTCTAGAGGCTCCTGACCCTTGCACCCTTGAGTCTTTCCTTGATAGAATCCCAATGGTCTTTAATGTTGTCATCCTTTCTCCTCATGGTTACTTTGCTCAAGATGATGTCTTGGGATACCCTGATACTGGAGGCCAGGTTGTTTACATCTTGGATCAAGTTCGTGCCTTGGAGAGCGAGATGCTCAGTCGCATTAAGAAACAAGGCTTGGATATCATCCCTCGCATTCTCATTATCACTCGTCTTCTCCCCGATGCAGTCGGAACTACTTGTGGCCAACGGCTTGAAAAGGTCTATGGAACTGAGCATTGCCACATTCTTCGAGTTCCCTTCAGAGATGAGAAGGGAATTGTTCGCAAGTGGATCTCACGATTTGAAGTCTGGCCATATCTAGAAACTTACACTGAGGATGTTGCTCATGAGCTTGCCAAAGAGTTGCAAAGCAAACCAGATCTGATTGTTGGAAACTACAGTGATGGAAACATTGTTGCCTCTTTGTTGGCACATAAATTAGGTGTCACTCAGTGTACCATTGCTCATGCACTCGAGAAGACTAAGTACCCCGAATCCGATATTTACTGGAAAAAATTCGAAGAGAAGTATCACTTCTCCTGCCAATTTACCGCTGATCTTTTCGCAATGAACCACACAGATTTCATCATCACTAGTACCTTCCAAGAGATTGCTGGAAGCAAGGATAAGGTTGGACAG

TATGAGAGTCACACTGCCTTTACTCTTCCAGGACTCTACCGTGTCGTGCACGGTATTGATGTCTTTGATCCAAAGTTCAACATTGTATCTCCAGGAGCTGATCAGACCATTTACTTCCCTTACACCGAAACTAGCCGCCGATTGACATCCTTCTACCCTGAAATCGAAGAGCTTCTTTACAGCTCAGTTGAGAATGAAGAGCACATATGTGTGCTGAAGGACCGCAACAAGCCAATTATCTTCACCATGGCAAGGTTGGACCGTGTGAAGAACATTACAGGACTTGTGGAGTGGTACGGCAAGAATGCCAAGCTTCGTGAGTTGGTGAACCTTGTTGTTGTGGCCGGAGACAGGAGGAAGGAGTCAAAGGACTTGGAAGAGATAGCTGAGATGAAGAAGATGTATGGCCTAATTGAGACCTACAAGTTGAATGGCCAATTCAGATGGATTTCCTCTCAGATGAACCGTGTCAGAAACGGAGAGCTGTACCGTGTGATTTGTGACACCAAGGGAGCTTTCGTGCAACCTGCCGTGTACGAAGCTTTCGGTTTGACAGTTGTTGAGGCCATGGCTACTGGATTGCCAACATTTGCAACTCTTAATGGTGGCCCTGCTGAGATCATTGTCCATGGCAAATCTGGATTCCACATTGATCCTTACCATGGTGACCGCGCTGCTGATCTCCTTGTTGAATTCTTTGAGAAGGTCAAAGCTGATCCATCTCACTGGGACAAGATCTCTCAAGGTGGTCTCCAACGTATTGAAGAGAA

GTATACATGGACAATTTACTCTCAGAGGCTTCTTACACTCACTGGTGTCTATGGCTTCTGGAAGCATGTGTCTAACCTCGACCGTCTTGAGAGCCGCCGCTATCTTGAGATGTTCTATGCTCTCAAGTACCGCAAATTGGCTGAGTCTGTGCCCCTAGCTGTTGAGTAATATTTGAGGCTTTGAAGAGTTAAATGAAGAAATGGAGAAACCGGCTTTGTTCTCTTATTTGGAGTCTTGTTTTGAGTTTTATAAATAAAAATGTCAATGATTTTGATTTTGTTAATTTAAGCTTTGGATAAAAAAATTGTCAATGTCTTTTTCTTTTGCATGATTTGAAATGTGATTGGGTGTTTTGCCCCATCAATTCAATTTTAAGCCGTGCCTTTATTGTTCATATTCTCCTTCAACTTTATTTTATCAAATTTCCAAAAAAAAAAAAAAAAAAAAAA

>MsSUS2

AACCATTTTGTCTACGATTTTCCGTGGCTATAAGTCTCTGCTTTTGTGCTCTGCTCAACACGTCGCACTAATTATCACTCTCTTAACGTTTTGGTTTTATCCAACTTAGAAATTCCAATTCCAATTCCATTTGAGTCCAACTTCTGGTCAGTTCTGCAGTCTAGAAGAACAACACTGTGATCATCGTTATGTCTACGCAAGCTAAGTATGTTCGAGTTCCCAGTATCAGAGACAGAGTTCAAGATACTCTTTCTGCTCACCGTAACGAACTCATTTCTCTCCTTTCCAGGTATGTGGCTCAGGGGAAAGGGATCTTGCAACCTCATAATTTGATTGATGAGCTTGAAAACATCCTTGGTCAGGAGGATCATCTTAAAGATGGCCCCTTTGGTGAAATTATCAAGTCTGCACAGGAAGCCATAGTTTTGCCTCCTTTTGTGGCAATAGCAGTTCGTCCAAGACCTGGTGTTTGGGAATATGTCCGTGTTAATGTCTTTGAACTCAACGTCGAGCAATTGAGCATTTCTGAATATCTCAGCTTCAAAGAAGAACTTGTAGATGGAAAGATTAATGATAATTTTGTACTGGAGCTAGATTTAGAGCCATTTAATGCATCATTTCCTCGTCCAACCCGTTCATCATCCATTGGCAATGGTGTGCAATTTCTCAACCGCCACCTCTCGTCAAATATGTTTCGCAACAAAGATTGCTTGGAACCCTTGCTTGATTTCCTCCGTGCTCACACATATAAAGGCCATGCGCTGATGTTAAATGATAGACTACAAAACATTTCCAAACTTCAGTCTGCTTTGGTCAAGGCCGAGGATCATCTTTCTAAGCTTGCGCCTGATACACTCTATTCTGAATTTGAATACTTATTACAAGGAATGGGGTTTGAGAGAGGTTGGGGTGATACTGCTGCGCGGGTATTAGAGACGATGCATCTGCTGTTGGATATTCTTCATGCCCCTGATCCTTCTACACTAGAGACTTTTCTTGGGAGAGTACCAATGGTGTTCAATGTTGTTATATTGTCCCCACATGGCTTCTTTGGGCAAGCCAATGTCCTGGGATTACCTGATACTGGCGGACAGGTTGTTTATATACTCGATCAAGTGCGTGCCCTTGAGAATGAGATGCTCGCTCGGATCCAGAAACAAGGACTTGATTTCACTCCTAGAATTTTAATTGTTACTAGGTTAATACCTGATGCAAAGGGGACAACATGCAACCAGAGACTAGAAAGAGTCAGTGGCACTGATTATACACATATTTTGCGTGTTCCATTCAGATCAGAAAAAGGAATTCTCCGTAAATGGATCTCAAGGTTTGATGTCTGGCCTTTCCTGGAAACTTATGCAGAGGATGTTGCTAGTGAAATTTCTGCCGAGTTACAATGTTATCCTGATTTCATCATAGGAAACTACAGTGATGGGAATCTTGTTGCATCTTTATTGGCTTACAAAATGGGAGTTACCCAGTGCACAATCGCACATGCATTGGAGAAGACAAAATATCCAGATTCGGATATATATTGGAAGAAATTTGAGGACAAATACCATTTTTCATGTCAGTTCACTGCTGACCTAATAGCCATGAATAATGCTGATTTTATCATCACCAGTACATACCAAGAGATTGCAGGAACGAAGAATACTGTTGGCCAGTATGAGAGTCACACTGCTTTTACTCTTCCTGGACTTTATAGGGTTGTCCATGGCATTGATGTTTTTGATCCCAAGTTCAATATTGTCTCTCCTGGAGCAGATTTGACAATATATTTCCCCTATTCTGAAAAGGAGAAAAGACTCACAGCTCTGCATGGTTCAATTGAAAAGCTATTGTATGATACCGAGCAGACCGATGAGTACATCGGTTCTCTGGCAGACCGGTCAAAGCCTATAATCTTCTCCATGGCAAGGCTTGACAGAGTTAAAAACATAACTGGTTTGGTAGAAAGCTATGCTAAGAACAGCAAATTGAGGGAACTGGTCAACCTTGTCGTAGTAGCTGGTTATATTGATGTGAAGAAGTCCAGTGACAGAGAAGAAATTGCAGAGATTGAGAAGATGCATGATCTCATGAAGCAGTATAATTTGAATGGCGAGTTTCGGTGGCTTACTGCTCAAACAAATAGAGCACGTAACGGGGAGCTGTATCGTTACATAGCAGATACAAAAGGCGCTTTCGTTCAGCCTGCTTTCTATGAAGCTTTTGGACTTACGGTCGTAGAGGCCATGACTTGTGGGCTCCCTACATTTGCCACTTGCCATGGTGGTCCTGCTGAGATCATCGAGAATGGTGTATCAGGATTTCATATTGATCCTTATCATCCCGACAAAGCTTCAGAGCTATTACTTGAATTTTTCCAAAAAAGTAAGGAGGATCCAAACCATTGGAACAAAATATCTGATGGAGGTCTTCAAAGAATTTTTGAAAGGTACACATGGAAGATCTATTCTGAAAGGCTTATGACCTTGGCGGGTGTTTATAGTTTCTGGAAATATGTTTCCAAACTTGAAAGGCGCGAAACTCGACGGTATCTTGAGATGTTCTACATTCTTAAGTTCCGCGATTTGGCAAATTCTGTTCCACTAGCAACGGATGACGCAAATTAACCAGCTGATGTACGGCAGCTCTATGTTCTTATCAAGGACTGACAGCAGACAATAAGAGTCTTGTATGTATATAAAAGTCTGCTTCTTGTGTTGTAAATTAATGTTTTTCTGAGGACTTGAGGAATTTCATGGAAGCTTGTTTGTGTTAGCTTTCATTATATCAGAAATGCCGAGTACTGGGTTTTGATGTCTCTTGTATTGTTTTCCCTGAGACAAGCTTAATGAGATACGGGAATAAGATTGGTAAGAACAAACCATGCTAAATCTTTCACCTAACTTTGAACAAGTGAGTTCATTATCATGTGAAGGTGTATTTGGGTCACGGATTCTCTACTTTTAGGCCGGAAAAGTCACTTTGATGTTGTATAATCTCTCTCCCGTATTGCATGCATTTAGGGATGGGAATAGGCCAGGCCGGTTTACAGGGGCCTACGGCCTAGCCTAC

>MsSUS3

ATGGGGATAGAAACAGAAGAAATTAAAATATGAAGAAACATAGAATGAGGAACTCTTCAATCTTTCTCCAAATTTCA CTATAAAAAGGGGTTCCAAATTCTTCACTCTTCACACATTCATTCCCCATTTCATTTACTTGTGGCTTTATCATTCTTTTGCGTTTGGTTTCTACCTTGCTTTCCCTCATTCTTTGCCACCCTTTTCTTGTCTCATTCAATATTTGCTCTATTTCTTCATTAGGTTCTTGCATTTCAATTACAACAAGTTGAAGTTTTAAGCTTTTGAAGCAATGGCTAGTCTTACCCGTTCAACTTCTCTCCGTGAGAGGTTTGATGAATCTCTCACTGCTCACAGGATTGAAATTTTGGCCCTTTTGTCAAGGATTGAAGCAAAGGGGAAGGGAATCCTACAACACCATCAAGTAATTGCTGAGTTTGAAGAAATCCCTGAGGAGAAGAGGCAAAAACTAGTTAATGGAGCCTTTGGAGAAGTTTTGAGATCTACTCAGGAAGCTGTAGTGTTGCCACCATTTGTCGCTCTTGCTGTTCGTCCAAGGCCTGGTGTTTGGGAGTATCTGAGAGTTGATGTGCACAGCCTTGTTGTCGATGAACTTCGTGCTGCCGAGTATCTCAAGTTCAAGGAGGAACTTGTTGAAGGAAGTTCTAATGGTAACTTTGTGCTTGAGTTGGACTTTGAACCATTCAACGCCTCTTTTCCTCGACCAACTCTGAACAAGTCGATTGGAAATGGCGTCGAGTTCCTCAATCGTCATCTTTCTGCTAAGCTTTTCCATGGCAAAGAAAGCTTGCAGCCACTGTTGGAGTTCCTCAGGCTTCACAACTATAACGGAAAGACTATGATGGTGAATGACAGAATTCAAAATCTGAATTCTCTTCAACATGTTTTGAGAACTGCAGAAGATTATCTTCGCACAATTGCTCCCGAAACACCCTACTCGGAATTTGAACACAAATTCCAGGATAGTGGTTTGGAGAGAGGGTGGGGTGACACGGCCGAGCGTGTCCTTGAAATGATCCAGCTTCTGTTGGATCTTCTCGAGGCACCTGATCCTTTCACCCTCGAGAAATTCCTTGGAAGAATCCCTATGGTCTTTAATGTTGTCATCCTTTCTCCACATGGTTACTTCGCCCAAGATAATGTCTTGGGATACCCTGATACCGGTGGACAGGTTGTGTACATTTTGGACCAAGTTCGAGCCTTGGAGAACGAGATGCTCAGACGTATCAAGCAACAAGGCTTGGATATCAAGCCTCGCATTCTCATTATCACCCGTCTTCTCCCTGATGCAGTGGGAACTACTTGTGGTGAACGTCTTGAGAAAGTATACGATACCGAACATTGCCACATTCTTCGAGTTCCATTTAGAACAGAGAAGGGAATTGTTCGCAAATGGATCTCAAGATTCGAAGTCTGGCCCTACCTTGAGACTTTCAGTGAGGATGTTGCCAATGAACTTGCAAAGGAGTTGGAAGGAAAACCTGATCTGATCGTCGGAAACTACAGTGATGGAAACATCGTTGCCTCTTTGTTAGCTCATAAATTAGGCGTAACTCAGTGTACTATTGCTCATGCACTCGAGAAGACCAAGTACCCAGAGTCTGAACTTTACTGGAAAAAATTCGAAGACAAGTATCACTTCTCATGCCAATTTACTGCTGATCTTTTTGCAATGAACCACACGATTTTATTATCACAA

>MsSUS5

CTAGGATTGCCAGACACTGGAGGCCAGGTGGTATACATTCTTGATCAAGTAAGAGCATTCGAGGAAGAGTTACTCCAAAAGATTGAGTTGCAAGGCCTTAATGTGAAGCCTCAAATTCTTGTGGTTACACGTCTAATACCGAATGCAAAAGGGACAACATGTAACCAAGAACTTGAACCTATCATCAAAACTAAGCATTCACACATTCTTAGAGTCCCTTTCTGGACAGAGAAGGGAATTCTATCCCAATGGGTGTCGCGTTTCGATATCTATCCTTATTTAGAAAGATTTGCTCAGGATTCTACTACCAAGATTCTTGAACTTATGGATGGAAAACCTGACCTCATTATTGGAAATTATACTGATGGAAACTTGGTTTCATCCTTGATGGCTTCTAAACTTGGTGTAACTCAGGCAACCATTGCTCATGCTTTGGAGAAAACAAAATATGAAGATTCAGATGCTAAATGGAATAGTTTTGAAGAAAAGTATCACTTTTCAAGTCAGTTCACAGCTGATCTAATCGCAATGAATTCCGCTGATTTTATCATAACTAGTACTTACCAAGAAATTGCTGGAAGCAAGGATAGGCCAGGACAATATGAAACTCACACTGCATTTACCATGCCGGGACTTTGTCGCGTAGTCTCTGGCATCAATGTATTTGATCCAAAGTTCAACATTGCTGCTCCAGGAGCTGATCAATCTGTCTACTTCCCTTTCACCGAGAAGAAGCAAAGATTGACCACTTTTCAACCTGCCATTGAAGAATTACTTTATAGTAAGGTTGAAAATGAAGAACACATTGGATTTTTGGAAGACAAGAAGAAACCAATAATATTCTCAATGGCAAGGCTTGACAAAGTGAAAAACATCAGCGGCCTAGTCGAGTGGTTTGCGAAGAACAAGAGACTAAGAAGTTTGGTAAATCTTGTAATTGTTGGCGGATTCTTTGATCCGTCAAAATCCAAAGATAGAGAAGAAACAGAAGAAATCAAGAAAATGCATTACTTGATGAAAGAGTACAAACTGCAAGGTCAGTTCAGATGGATTGCGGCGCAAACTGATAGATATCGCAACGGAGAGCTTTACCGATGCATCGCGGATACAAAAGGAGCATTTGTTCAACCAGCATTGTATGAAGCTTTTGGTCTAACAGTGATTGAGGCAATGAACTGTGGATTACCAACTTTTGCAACAAACCAAGGTGGTCCAGCTGAAATTATAATTGATGGTGTCTCAGGTTTTCATATTGATCCTCATAATGGAGATGAATCAATCAACAAGGTTTCTGAATTTTTTGAGAAGTGCAAGACTAATCCTGAACATTGGAACATAGTTTCTAAAGCTGGTCTTCAAAGAATCAATGAATGCTATACATGGAAGATATATGCAAACAAAGTGTTGAATATGGGATCAATTTATGGGTTTTGGAGAAAGTTGAACAAAGAACAAAAGTTGGCAAAGGAAAGATACATACAAATGTTTTATAATCTTCAATTTAGGAACTTGGCAAGAAAGGTACCAATTCCTAAGGTACCCCAAGAACCTCAACCAATGTCAACAACTCCTGCCAAAAAAGCAGAAGTTACTTCCATGGGGCATGATGCAGTACAAGCAAAAGCTGAAGTACCTCAAACCCAGTTGTCATCATTACCACCCAAAATTGAATCTAAGCTAACATCAAGGGGTGAATCATCAAGTAAGGAACTAGCAGCAAAACAAAGTGGTGATCTTTATTCTGGATTACGTTGGTTGCTTCCAGGAATTGCTTTTATGTTCATCATTCATTATCTCTCAAAGTATTTGGAACATTTATTCACACGGGAGCAATAATCTCTAAGATGAAATCACAATCATATGTGGATGTAATTTTAGTTTTTACTTATTTGTATCTTTACCTTTTTATTTTAACTTTATTGTAAATGATTTCGGTGTACATAAGAAGTGTCATTTCCACAATACACATTTGGAATTAAATAGTGGCATTTGAAATCAATATATTA
