# Supplementary material for: Transgene silencing of sucrose synthase in alfalfa (Medicago sativa L.) stem vascular tissue suggests a role for invertase in cell wall cellulose synthesis
Source: BMC Plant Biol. 2015 Dec 1;15:283. doi: 10.1186/s12870-015-0649-4 (PMC4666122; doi:10.1186/s12870-015-0649-4)

**Additional file 2**. Phylogenetic analysis of *SUS* genes in alfalfa (Ms), *Medicago truncatula* (Mt), *Arabidopsis* (At)*,* and *Pisum sativum* (Ps)*.*

The tree is drawn to scale, with branch lengths in the same units as those of the evolutionary distances used to infer the phylogenetic tree. GenBank accessions for the *Arabidopsis thaliana* isoforms 1, 2, 3, 4, 5, and 6 are BAH19538, Q00917, Q9M111, Q9LXL5, F4K5W8, and Q9FX32, respectively. GenBank accessions for *Pisum sativum* isoforms 1, 2 and 3 are AAC28107, O24301 and CAC32462, respectively. The *Medicago truncatula* isoforms were obtained from the *Medicago truncatula* 11.0 gene index (compbio.dfci.harvard.edu/tgi/) with isoforms 1, 2, 3, and 5 encoded by MtTC172525, MtTC193395, MtTC189980, and MtTC65865, respectively. *Medicago sativa* *SUS1* sequence can be found as GenBank AF049487. The remaining isoform sequences were mined from alfalfa RNA-seq data (<http://plantgrn.noble.org/AGED/>), Additional file 1).


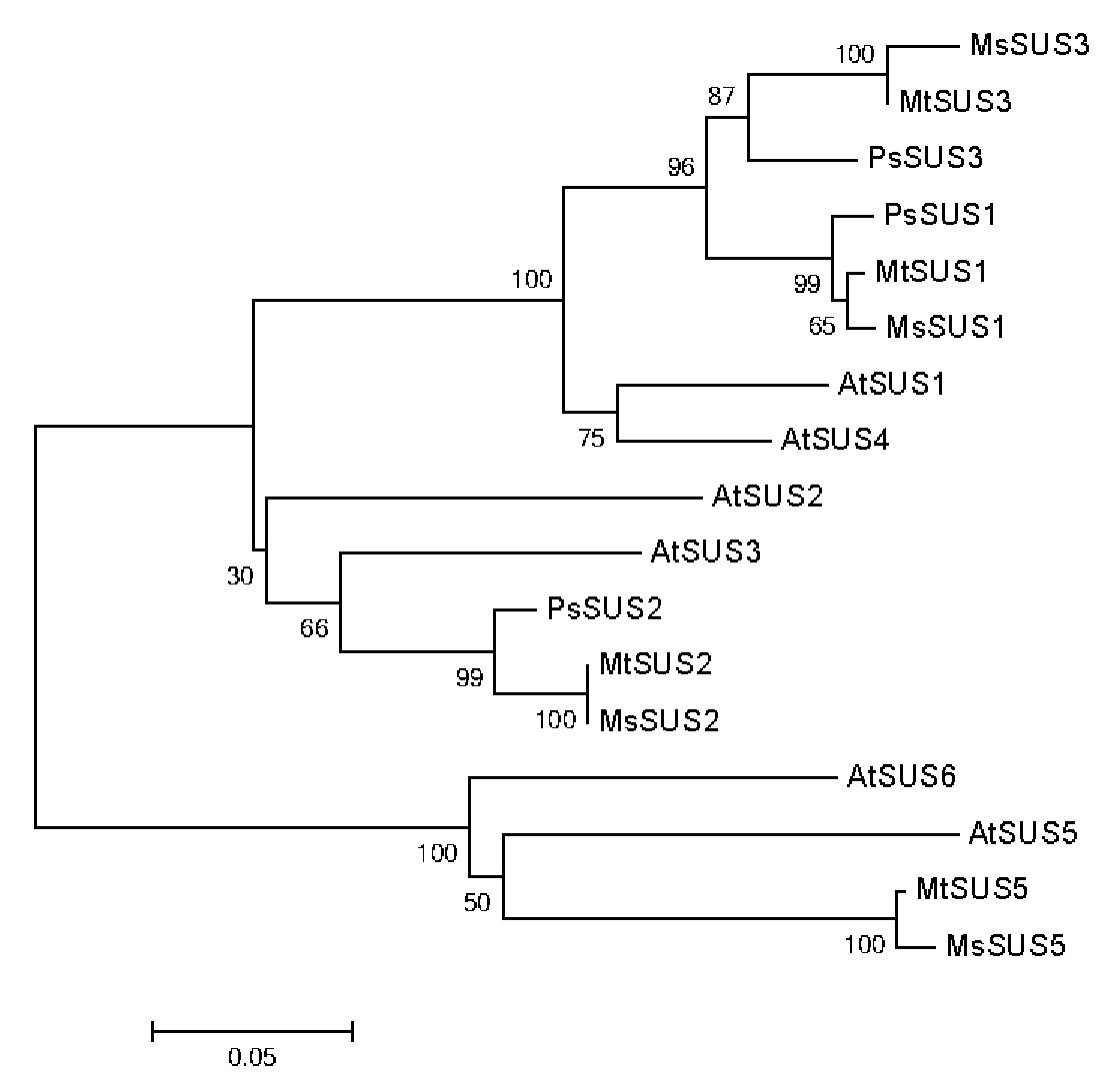

Supplement: Additional file 2: — Phylogenetic analysis of SUS genes in alfalfa (Ms), Medicago truncatula (Mt), Arabidopsis thaliana (At) and, Pisum sativum (Ps). (DOCX 181 kb) [file 12870_2015_649_MOESM2_ESM.docx]
