# Supplementary material for: Transgene silencing of sucrose synthase in alfalfa (Medicago sativa L.) stem vascular tissue suggests a role for invertase in cell wall cellulose synthesis
Source: BMC Plant Biol. 2015 Dec 1;15:283. doi: 10.1186/s12870-015-0649-4 (PMC4666122; doi:10.1186/s12870-015-0649-4)
Supplement: Additional file 3: — Primer sequences used for qPCR. (DOCX 62 kb) [file 12870_2015_649_MOESM3_ESM.docx]

**Additional file 3.** Primer sequences used for qRT-PCR.

| **Gene** | **Primer Pairs** | **Amplicon Size** |
| --- | --- | --- |
| *MsSUS1*  *MsSUS2*  *MsSUS3*  *MsSUS5* | 5'-GACCGCAACAAGCCAATTAT-3'  5'-AACTCACGAAGCTTGGCATT-3'  5’-GAGATGCTCGCTCGGATCCAG-3’  5’-GTGTATAATCAGTGCCACTGACTC-3’  5’-GGTGAACGTCTTGAGAAAGTATAC-3’  5’-CATCCTCACTGAAAGTCTCAAGG-3’  5’-GTTGCAAGGCCTTAATGTGAAGCC-3’  5’-GTGAATGCTTAGTTTTGATGATAGG-3’ | 104 bp  136 bp  139 bp    116 bp |
| *MsACT2* | 5’-CCACATGCCATCCTTCGTTT-3’  5’-TGTCACGGACAATTTCCCG-3’ | 121 bp |
