# Supplementary material for: Transgene silencing of sucrose synthase in alfalfa (Medicago sativa L.) stem vascular tissue suggests a role for invertase in cell wall cellulose synthesis
Source: BMC Plant Biol. 2015 Dec 1;15:283. doi: 10.1186/s12870-015-0649-4 (PMC4666122; doi:10.1186/s12870-015-0649-4)
Supplement: Additional file 4: — Cell wall content of control (M22) and the PEPC7-P4::MsSUS transformants (M17, M18). (DOCX 64 kb) [file 12870_2015_649_MOESM4_ESM.docx]

**Additional file 4.**

Cell wall content of control (M22) and the *PEPC7-P4::MsSUS1* transformants (M17, M18).

| Trait |  | | |
| --- | --- | --- | --- |
|  | M22 | M17 | M18 |
| Concentration | - - - - - - - - - - - - - - - g/kg dry matter - - - - - - - - - - - - - - - | | |
| Cell wall | 684 (6.1)^a^ | 675 (10.4) | *665 (7.6) |
|  |  |  |  |
| Composition | - - - - - - - - - - - - - - - g/kg cell wall - - - - - - - - - - - - - - - | | |
| Klason lignin | 231 (7.1) | 222 (4.0) | 214 (1.9) |
| Uronic acids | 134 (4.3) | 133 (2.6) | 124 (8.3) |
| Rhamnose | 10.1 (0.1) | 10.5 (0.4) | 9.8 (0.3) |
| Fucose | 1.5 (0.01) | 1.5 (0.05) | 1.2 (0.1) |
| Arabinose | 39.5 (1.5) | 40.6 (1.5) | 37.7 (2.2) |
| Xylose | 156 (5.0) | 158 (3.6) | 156 (2.7) |
| Mannose | 22.7 (0.1) | 24.8 (0.3) | 25.0 (0.3) |
| Galactose | 24.2 (0.4) | 26.0 (0.5) | *25.6 (0.5) |
| Glucose | 383 (0.6) | 384 (9.6) | 408 (9.0) |

^a^ Values are means ± standard error in parenthesis, n = 4. Statistically significant differences (*P* < 0.05) as determined by analysis of variance between the control and M17or M18 are indicated by an asterisk.
